# Immunogenic properties of amyloid beta oligomers

**DOI:** 10.1186/1423-0127-20-10

**Published:** 2013-02-22

**Authors:** Indre Dalgediene, Rita Lasickiene, Rima Budvytyte, Gintaras Valincius, Ramune Morkuniene, Vilmante Borutaite, Aurelija Zvirbliene

**Affiliations:** 1 Institute of Biotechnology, Vilnius University, V. Graiciuno str. 8, LT-02241, Vilnius, Lithuania; 2 Institute of Biochemistry, Vilnius University, Mokslininku str. 12, LT-08662, Vilnius, Lithuania; 3 Institute of Neurosciences, Lithuanian University of Health Sciences, Eiveniu str. 4, LT-50009, Kaunas, Lithuania

**Keywords:** Alzheimer’s disease (AD), Amyloid beta (Aβ), Neurotoxicity, Immunogenicity, Neurotoxic oligomers, Epitope mapping

## Abstract

**Background:**

The central molecule in the pathogenesis of Alzheimer’s disease (AD) is believed to be a small-sized polypeptide – beta amyloid (Aβ) which has an ability to assemble spontaneously into oligomers. Various studies concerning therapeutic and prophylactic approaches for AD are based on the immunotherapy using antibodies against Aβ. It has been suggested that either active immunization with Aβ or passive immunization with anti-Aβ antibodies might help to prevent or reduce the symptoms of the disease. However, knowledge on the mechanisms of Aβ-induced immune response is rather limited. Previous research on Aβ1-42 oligomers in rat brain cultures showed that the neurotoxicity of these oligomers considerably depends on their size. In the current study, we evaluated the dependence of immunogenicity of Aβ1-42 oligomers on the size of oligomeric particles and identified the immunodominant epitopes of the oligomers.

**Results:**

Mice were immunized with various Aβ1-42 oligomers. The analysis of serum antibodies revealed that small Aβ1-42 oligomers (1–2 nm in size) are highly immunogenic. They induced predominantly IgG2b and IgG2a responses. In contrast, larger Aβ1-42 oligomers and monomers induced weaker IgG response in immunized mice. The monoclonal antibody against 1–2 nm Aβ1-42 oligomers was generated and used for antigenic characterization of Aβ1-42 oligomers. Epitope mapping of both monoclonal and polyclonal antibodies demonstrated that the main immunodominant region of the 1–2 nm Aβ1-42 oligomers is located at the amino-terminus (N-terminus) of the peptide, between amino acids 1 and 19.

**Conclusions:**

Small Aβ1-42 oligomers of size 1–2 nm induce the strongest immune response in mice. The N-terminus of Aβ1-42 oligomers represents an immunodominant region which indicates its surface localization and accessibility to the B cells. The results of the current study may be important for further development of Aβ-based vaccination and immunotherapy strategies.

## Background

Alzheimer’s disease (AD) is the most common progressive neurodegenerative disease in elderly people. The disease manifests as decline in cognitive functions and memory loss followed by changes in personality and mental state. It is known that while the disease evolves the main changes take place in parts of the brain responsible for the cognitive functions and memory. One of the central molecules involved in the pathogenesis of AD is thought to be a small sized polypeptide – beta amyloid (Aβ) which has an ability to assemble spontaneously into oligomers [1]. It has been shown that these oligomers depending on their size and shape have different neurotoxic effects on rat brain cell cultures [2]. Several therapeutic and prophylactic approaches based on the anti-amyloid immunotherapy have been suggested for AD and clinical trials have been started with either active immunization with Aβ protein or passive immunization with anti-Aβ antibodies. However, acute brain tissue inflammation while using these treatments has been reported and several clinical trials were cancelled due to this side effect [3-5]. Therefore, better understanding of the antigenic properties of Aβ and its effects on the functions of immune cells may lead to the improvement of the anti-amyloid immunotherapy.

In the current study we investigated the immunological and antigenic properties of Aβ1-42 oligomers. We demonstrated that various Aβ1-42 oligomers differ in their capacity to induce antibody response in experimental mice. We also employed the monoclonal and polyclonal antibodies generated against Aβ1-42 oligomers to identify their immunodominant epitopes.

## Methods

### Aβ1-42 oligomers

All Aβ1-42 oligomers used in this study were prepared and characterized as described previously [2,6,7]. Synthetic Aβ1-42 peptide was purchased from American Peptide Company (Sunnyvale, California, USA). Millipore purified water (18.2 MΩ cm) was used throughout the work.

Briefly, broad size range (1–15 nm in size) Aβ1-42 oligomers were prepared by dissolving 0.3 mg of Aβ1-42 and incubating it in 130 μl hexafluoroisopropanol (HFIP) for 20 min in 1.5 ml vials, followed by addition of 900 ml H_2_O and 20 min incubation in the resulting HFIP/water mixture. Subsequently, the samples were centrifuged at 12,000 g for 15 min. The solvent was evaporated from resulting supernatant under nitrogen stream for 5 min. Then the samples were incubated in closed vials for 24 h with constant stirring at room temperature.

The protocol described above was modified for producing smaller Aβ1-42 oligomeric particles. 1 mg of peptide was dissolved in 400 μl HFIP and incubated for 30–60 min at room temperature. 100 μl of the resulting seedless solution was added to 900 μl H_2_O in a siliconized vial. After 10–20 min of incubation at room temperature the samples were centrifuged for 15 min at 12,000 g, the supernatant was transferred to a new siliconized tube and HFIP was evaporated in ambient air for 1 h. Then the samples were incubated in closed vials for 24 h in a thermostatic bath, at +20°C. The fraction of Aβ1-42 oligomeric particles of 1–2 nm size was produced by this protocol.

To generate larger, typically 5–10 nm Aβ1-42 oligomers, after the centrifugation the supernatant was transferred to a nonsiliconized vial and gently purged with nitrogen for 7 min. Then the preparation was stirred in the same vial at ~ 500 rpm for 24 h using a magnetic Teflon-coated stirring bar.

Finally, the Aβ1-42 monomers were formed by dissolving Aβ1-42 peptide in Millipore purified water immediately before the experiments. All solutions of prepared Aβ1-42 oligomers were stored at −20°C no longer than for 24 h.

Shortly, to assess the size and morphology of the preparations of Aβ1-42 oligomers, atomic force microscopy (AFM; Agilent 5500, Santa Clara, California, USA) was used in the tapping mode. According to the manufacturer’s recommendations, the probe tip diameters were between 16 and 20 nm. About 20 μl of a 10 μM Aβ1-42 solution was spotted on freshly cleaved mica (SPI Supplies, West Chester, Pennsylvania, USA), incubated at room temperature for 10 min and rinsed with Millipore purified water, then blown dry with a nitrogen stream. Images were acquired at scan rates between 0.5 and 1 Hz with the drive amplitude and force kept to a minimum. The particle size was estimated by measuring the profile of the sample within the sample plane.

### Recombinant antigens

Recombinant proteins used for the analysis of antibody specificity by ELISA: hamster polyomavirus major capsid protein VP1 (VP1) and human metapneumovirus (hMPV) nucleocapsid (N) protein (hMPV N) were described previously [8,9].

Recombinant Aβ1-40 protein fused with thioredoxin (Trx-Aβ1-40) and expressed in *E*. *coli* was used for the analysis of antibody reactivity by Western blot. Shortly, thioredoxin gene was fused with Aβ1-40 gene at its N-terminus and cloned into expression vector pET3a. Fused protein Trx-Aβ1-40 was expressed in *E*. *coli* strain DH5α and purified under denaturing conditions using Ni chelating column.

### Immunization of mice and generation of monoclonal and polyclonal antibodies

BALB/c mice were bred and maintained in an animal facility at the Department of Immunology of the Centre for Innovative Medicine (Vilnius, Lithuania). The groups of 4 female mice aged 6–8 weeks per each antigen were immunized with Aβ1-42 broad size range oligomers, 1–2 nm Aβ1-42 oligomers, 5–10 nm Aβ1-42 oligomers and Aβ1-42 monomers (non-treated peptide). Control group of BALB/c mice (n = 4) received PBS injections. All injections were subcutaneous. The dose was 50 μg of oligomers or peptide per mouse. For the primary immunizations the antigens were emulsified in complete Freund’s adjuvant (Sigma-Aldrich, St. Louis, Missouri, USA). The second immunization followed on day 28 with the antigens dissolved in PBS. Antiserum samples were collected on day 14 after the first and second immunizations and tested by an indirect enzyme-linked immunosorbent assay (ELISA) for the presence of IgG antibodies specific to Aβ1-42 oligomers and the monomers. The spleen cells of the mouse with the highest antibody titre were used for the generation of hybridomas [10]. Three days after the boost immunization the spleen cells of the mouse were fused with Sp2/0-Ag14 mouse myeloma cells using polyethylene glycol 1500 (PEG/DMSO solution, HybriMax, Sigma-Aldrich). Hybrid cells were selected in growth medium supplemented with hypoxantine, aminopterin and thymidine (50× HAT media supplement, Sigma-Aldrich). Samples of supernatant from wells with viable clones were screened by an indirect ELISA. Hybridomas secreting Aβ1-42 specific antibodies were subcloned twice by a limiting dilution method. Hybridoma cells were maintained in complete Dulbecco's modified Eagle's medium (DMEM, Biochrom, Berlin, Germany) containing 15 ; fetal calf serum (Biochrom) and antibiotics. Antibodies were isotyped using Monoclonal Antibody Isotyping Kit I (HRP/ABTS) (Pierce Biotechnology, Rockford, Illinois, USA) in accordance with the manufacturer's protocol. All procedures involving experimental mice were performed under controlled laboratory conditions in strict accordance with the Lithuanian and European legislation.

### Indirect enzyme-linked immunosorbent assay (ELISA) analysis for anti-Aβ1-42 antibodies

Microtiter plates (Nunc MaxiSorp, Nunc, Roskilde, Denmark) were coated with 100 μl/well of either Aβ1-42 oligomers or Aβ1-42 peptide dissolved in the coating buffer (0.05 M sodium carbonate, pH 9.5) to a concentration of 5 μg/ml. For the coating with Aβ1-42 oligomers the plates were incubated overnight at +4°C. The Aβ1-42 peptide was dried in the plates by incubating overnight at +37°C. The coated plates were blocked with 250 μl/well of PBS with 2 ; BSA for 1 h at room temperature (RT). Then plates were rinsed twice with PBST (PBS with 0.1 ; Tween-20). Antiserum samples, hybridoma growth medium or polyclonal antibodies were diluted in PBST, added to the wells (100 μl/well) and incubated for 1 h at RT. The plates were then incubated for 1 h with Goat Anti-Mouse IgG (H+L)-HRP Conjugate (Bio-Rad, Hercules, California, USA) diluted 1:5000 in PBST. The enzymatic reaction was visualized by the addition of 100 μl of “NeA-Blue” TMB solution (Clinical Science Products, Mansfield, Massachusetts, USA) to each well. The reaction was stopped by adding 50 μl/well of 10 ; sulphuric acid solution. The optical density (OD) was measured at 450 nm (reference filter 620 nm) in a microplate reader (Sunrise Tecan, Männedorf, Switzerland).

### SDS-PAGE and western blot analysis

The samples of recombinant fused protein Trx-Aβ1-40, *E*. *coli* DH5α lysate and HeLa lysate were boiled in a reducing sample buffer and separated in 15 ; polyacrylamide gel electrophoresis (PAGE) in SDS–Tris–glycine buffer. Proteins were visualized by staining with Coomassie Brilliant blue (Sigma-Aldrich). The proteins from the unstained SDS-PAGE gel were blotted onto Roti®-PVDF membrane (Carl Roth, Karlsruhe, Germany) by semidry electro-transfer. The membrane was blocked with 2 ; milk powder in PBS for 2 h at RT and rinsed with PBST. The membrane was then incubated for 1 h at RT with primary antibodies at working dilution in PBST with 2 ; milk powder and subsequently incubated with Goat Anti-Mouse IgG (H+L)-HRP Conjugate (Bio-Rad) diluted 1:4000 in PBST with 2 ; milk powder. The enzymatic reaction was developed using 4-chloro-1-naphtol and H_2_O_2_ (Fluka, Milwaukee, Wisconsin, USA). For the analysis of antisera, they were diluted 1:1000 in PBST. For the analysis of monoclonal antibodies, undiluted hybridoma supernatant was used.

### Characterization of Aβ1-42 epitopes recognized by polyclonal and monoclonal antibodies

For the epitope mapping, the collection of synthetic, overlapping, Aβ1-42 spanning, 13 amino acids (aa) in length and linked with biotin-SGSG at the N-terminus peptides was used (Metabion, Martinsried, Germany). Microtiter plates (Nunc MaxiSorp, Nunc) were coated with streptavidin dissolved in deionized H_2_O (2 μg/ml) by incubating overnight at +37°C. The coated plates were blocked with 2 ; BSA in PBS. Synthetic peptides with biotin were added to the wells diluted in PBST at concentrations recommended by the manufacturer and incubated for 1 h at RT. Hybridoma supernatant (undiluted) and polyclonal antibodies (diluted 1:1000 in PBST) were added to the wells (100 μl/well) and incubated for 1 h at RT. The plates were incubated for 1 h with Goat Anti-Mouse IgG (H+L)-HRP Conjugate (Bio-Rad) diluted 1:5000 in PBST. The enzymatic reaction was visualized with TMB as described above.

## Results

### Immune response to Aβ1-42 oligomers in mice

**Figure 1:**
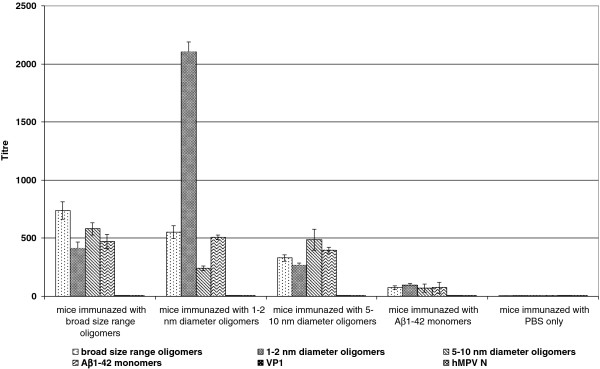
**Titres of Aβ1-42 specific IgG in the antisera of immunized mice.** The immunogenicity of Aβ1-42 oligomers was determined by an indirect ELISA using mouse antisera raised against Aβ1-42 oligomers. The microtiter plate was coated with various Aβ1-42 oligomers, Aβ1-42 peptide (monomers) or irrelevant antigens and subsequently incubated with the antisera collected on the 14^th^ day after the second immunization.

To investigate the immunogenicity of various Aβ1-42 oligomer forms, four groups of BALB/c mice were exploited. Each group of mice was injected with the same doses (50 μg per mouse) of the antigens: broad size range Aβ1-42 oligomers, Aβ1-42 oligomers with particle size of 1–2 nm, Aβ1-42 oligomers with 5–10 nm particle size and Aβ1-42 monomers. The levels of antigen-specific IgG antibodies in the antiserum samples were estimated by calculating the antibody titres in ELISA against the antigens used for the immunization. After the first immunization, the titres of antigen-specific IgG antibodies in all groups of immunized mice did not exceed 1:100 (data not shown). Although the adjuvant was used for the primary immunization to enhance the immune response, the adjuvant might have also disrupted the oligomeric structure of the antigens. After the secondary immunization applied without an adjuvant there was a significant difference in antibody titres between all groups of immunized mice (Figure 1). The highest antibody response was observed in mice immunized with 1–2 nm Aβ1-42 oligomers. The titres of antigen-specific IgG were almost 3 times higher in this group of mice as compared to that immunized with broad size range oligomers (1:2000 and 1:700, respectively). In contrast, the titres of antigen-specific IgG in the group of mice immunized with 5–10 nm Aβ1-42 oligomers did not exceed 1:500. Thus, the series of the strongest Aβ1-42 immunogens varied from 1–2 nm oligomers to the broad size range oligomers and 5–10 nm oligomers. As expected, Aβ1-42 monomers demonstrated the weakest immunogenic properties giving antibody titres about 1:200 (Figure 1). Moreover, antisera raised against 1–2 nm Aβ1-42 oligomers showed in ELISA 4 times lower titres with Aβ1-42 monomers as compared to 1–2 nm Aβ1-42 oligomers: 1:500 and 1:2000, respectively. Antisera collected from the control group of mice injected with PBS alone showed only background level activity with antibody titres less than 1:20. These results demonstrated that Aβ1-42 oligomeric structure is important in the activation of B cells and induction of the antibody response. To evaluate the involvement of helper T cells in B cell activation, the subclass distribution of antigen-specific IgG antibodies in the pool of antisera of mice immunized with 1–2 nm Aβ1-42 oligomers was analysed. The majority of the IgG antibodies (57 ;) raised against 1–2 nm Aβ1-42 oligomers were of IgG2a isotype (Table 1). In contrast, antigen-specific antibodies of IgG1 and IgG3 subtypes were presented to a less extent (19 ; and 2 ; of total IgG, respectively).

**Table 1:** Distribution of the subtypes of IgG antibodies raised against 1–2 nm Aβ1-42 oligomers, expressed in percents of total Aβ1-42 specific IgG

**Subtype**	**Percents of total Aβ1-42 specific IgG, ;**
IgG1	19
IgG2a	57
IgG2b	22
IgG3	2

### Generation of monoclonal antibodies against Aβ1-42 oligomers

**Figure 2:**
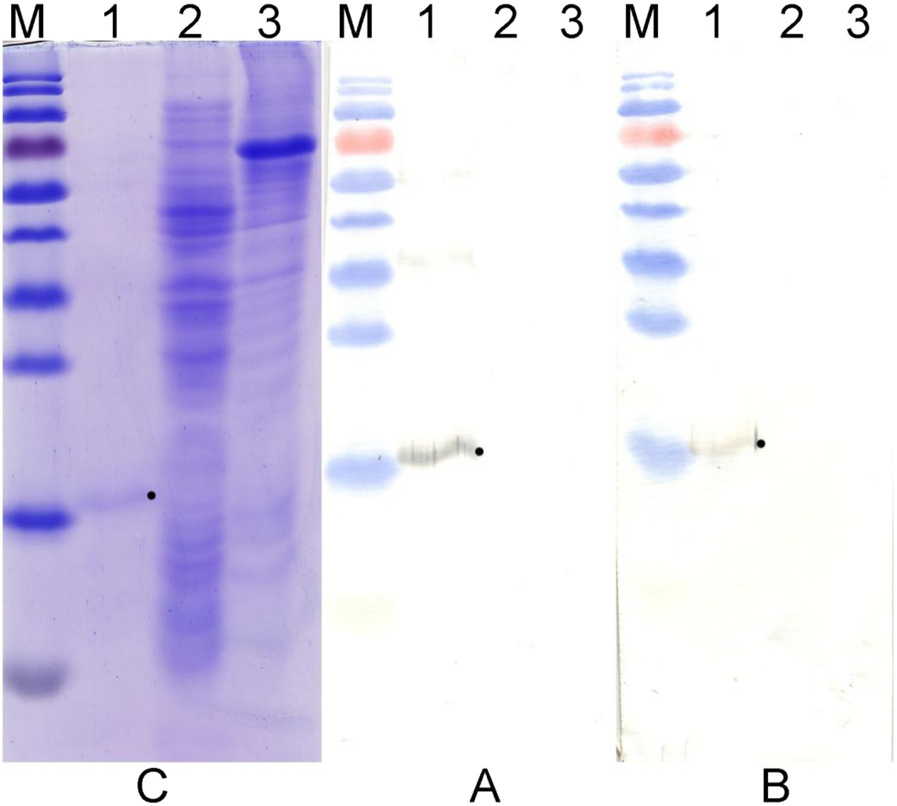
**Reactivity of monoclonal antibody 11E12 (A) and antiserum of the mouse immunized with 1–2 nm Aβ1-42 oligomers (B) in Western blot.C** – Coomasie blue-stained gel. Lane 1, recombinant fused protein Trx-Aβ1-40; lane 2, *E*. *coli* DH5α cells lysate; lane 3, HeLa cells lysate. Lane M, prestained protein molecular weight marker: 130, 100, 70, 55, 40, 25, 15 kDa (Fermentas/Thermo Scientific, Vilnius, Lithuania). The position of recombinant fused protein Trx-Aβ1-40 (molecular weight in a range of 16–20 kDa) is indicated by a dot.

Spleen cells of mice with the highest antibody titre induced by 1–2 nm Aβ1-42 oligomers were used to generate the monoclonal antibodies (MAbs). One stable hybridoma clone 11E12 producing Aβ1-42-specific MAb of IgG2b subtype was generated. An indirect ELISA with different Aβ1-42 oligomers and the monomers revealed a strong reactivity of the MAb 11E12 with 1–2 nm Aβ1-42 oligomers: titres of antigen-specific antibodies in the hybridoma supernatant were 1:2000. In contrast, the reactivity of the MAb 11E12 with 5–10 nm oligomers and the monomeric peptide was significantly lower: titres in ELISA were 1:80 and 1:50, respectively (data not shown). The specificity of the MAb to the Aβ1-42 sequence was also confirmed by Western blot analysis of recombinant fused protein Trx-Aβ1-40. The MAb 11E12 reacted specifically with recombinant Trx-Aβ1-40 but did not react with *E*. *coli* DH5α lysate and HeLa cell lysate used as negative controls ( 2A). Antiserum of the mouse immunized with 1–2 nm Aβ1-42 oligomers and used for the generation of monoclonal antibody 11E12 also showed reactivity with recombinant fused protein Trx-Aβ1-40 (Figure 2B). In contrast, pools of antisera raised against broad size range Aβ1-42 oligomers, 5–10 nm Aβ1-42 oligomers and Aβ1-42 monomers showed week or no reactivity with recombinant fused protein Trx-Aβ1-40 (data not shown).

### Mapping of Aβ1-42 epitopes recognized by polyclonal and monoclonal antibodies

To investigate the antigenic structure of Aβ1-42 oligomers, we have employed a series of overlapping 13 aa-long synthetic peptides spanning the Aβ1-42 sequence (Table 2). The MAb 11E12 reacted exclusively with the peptide representing the N-terminal sequence (aa 1–13, Table 2). Antiserum of the mouse immunized with 1–2 nm Aβ1-42 oligomers and used for the generation of monoclonal antibody 11E12 showed strong reactivity exclusively with peptides #1 and #2 representing the N-terminus of Aβ1-42 protein (aa 1–19). Pools of antisera raised against the broad size range Aβ1-42 oligomers and 5–10 nm Aβ1-42 oligomers showed a week positive reaction also with the N-terminal peptide #1 and did not show any reactivity with other peptides. Thus, regardless of the form of Aβ1-42 oligomers the N-terminus was identified as an immunodominant region of Aβ1-42 oligomers.

**Table 2:** Epitope mapping of Aβ1-42 monoclonal antibody (MAb) 11E12 and polyclonal antisera based on their immunoreactivity with overlapping peptides spanning Aβ1-42 sequence

**Peptide No.**	**aa sequence**	**The reactivity of peptides with different antibodies in ELISA:**			
		**MAb 11E12**	**Antisera to broad size range oligomers**	**Antisera to 1–2 nm oligomers**	**Antisera to 5–10 nm oligomers**
1 (aa 1–13)	DAEFRHDSGYEVH	**++**	**+**	**++**	**+**
2 (aa 7–19)	DSGYEVHHQKLVF	-	-	+	-
3 (aa 13–25)	HHQKLVFFAEDVG	-	-	-	-
4 (aa 19–31)	FFAEDVGSNKGAI	-	-	-	-
5 (aa 25–37)	GSNKGAIIGLMVG	-	-	-	-
6 (aa 31–42)	IIGLMVGGVVIA	-	-	-	-

Every next peptide was starting with the same 7 aa from the previous one. The last peptide was 12 aa in length. The interaction between peptides and antibodies in an indirect ELISA is expressed as “++” – strong (OD > 1.0), “+” – weak (1.0 > OD > 0.25) and “-“– none (OD < 0.25).

## Discussion

The attempts to use active immunization with Aβ or passive immunization with anti-Aβ antibodies represent promising new strategies in the immunotherapy of AD. However, the results of previous preclinical and clinical studies of AD immunotherapy were controversial [3,5]. In some cases acute brain inflammation occurred [3]. A number of questions on the origin and mechanism(s) of these unwanted side effects still remains unanswered. It is not yet known what impact on the pattern of the immune response various aggregate forms of Aβ may have. To elucidate the mechanisms of anti-Aβ immune response, naturally occurring antibodies against Aβ have been investigated [11]. It is also possible to focus on various artificial Aβ aggregate forms and gain information on their immunogenic and antigenic properties using laboratory animal models.

In the current study, we have investigated the immunogenicity of various Aβ1-42 oligomers including the small-sized oligomers that were shown to be highly neurotoxic *in vitro*[2,12]. The analysis of the humoral immune response in mice immunized with different Aβ1-42 oligomers revealed that their immunogenicity strongly depends on the size of oligomeric particles so that the smallest Aβ1-42 oligomers with particle size of 1–2 nm (corresponding to dimers – pentamers [2]) were the strongest immunogens in comparison with 5–10 nm oligomers or broad size range oligomers. Moreover, the analysis of IgG subtypes in the sera of immunized mice revealed IgG2a as the major IgG subclass of Aβ-specific antibodies thus suggesting the predominant role of Th1 cells in B cell activation (Table 1). On the other hand, it cannot be excluded that IgG subtype distribution was influenced by the use of adjuvant since Aβ1-42 oligomers were non-immunogenic in mice when applied without it. Previous studies on the neurotoxicity of different Aβ1-42 oligomers in rat brain cell cultures revealed that the most neurotoxic Aβ1-42 oligomers are of size 1–2 nm [2]. Thus, the smallest 1–2 nm Aβ1-42 oligomers possess both high neurotoxicity and immunogenicity. The nature of the link between those two properties of the smallest 1–2 nm Aβ1-42 oligomers remains still to be elucidated.

Our study demonstrated that the N-terminal part of the Aβ1-42 oligomers was accessible to B cells in active immunization and induced the development of Aβ1-42 specific antibodies. The obtained data on the surface-exposure of the N-terminal part of Aβ1-42 oligomers are in line with previous studies proposing that the assembly of oligomers starts at the middle and carboxy-terminal (C-terminal) parts of the Aβ [13,14]. Therefore, active immunization with Aβ oligomers may lead to generation of N-terminus specific antibodies. The immunogenicity of the N-terminal part of Aβ1-42 oligomers also indicates that the oligomers tend to maintain their structural features during the interactions with B cells. This presumption is based on the fact that both monoclonal antibody 11E12 and polyclonal antisera raised against 1–2 nm Aβ1-42 oligomers showed higher reactivity with Aβ1-42 oligomers as compared to Aβ1-42 monomers. On the other hand, both the antibody 11E12 and polyclonal antisera were reactive with synthetic peptides representing N-terminal linear epitopes of Aβ1-42 thus suggesting that conformational epitopes were present but not immunodominant in Aβ1-42 oligomers. Previous studies have demonstrated that passive immunization with a monoclonal antibody directed against the central domain of Aβ peptide (13–28 aa) was effective in reducing brain Aβ burden by promoting CNS and plasma Aβ clearance in mice [15,16]. Clinical trials on active immunization with Aβ1-42 peptide (AN1792) revealed that plaque load has decreased, however this decrease was insufficient to improve the cognitive functions of AD patients [5]. It has been shown that the peptide AN1792 induced antibodies against the N-terminus of Aβ protein (1–8 aa) [17]. Thus, the Aβ oligomers used in our study resemble immunogenic properties of those explored in other immunization studies. In contrast, naturally occurring anti-Aβ antibodies have been shown to be mainly directed to the middle and/or C-terminal epitopes of Aβ protein and to inhibit the tendency of Aβ protein to fibrillate thereby blocking its neurotoxicity [18-20]. The latest studies have shown that these naturally occurring antibodies bind to early oligomerization products and interfere with this process [11]. In contrast, antibodies raised against the N-terminus of Aβ protein may not necessarily interfere with the oligomerization of Aβ protein. These studies suggest that further assessments should be made for the development of new immunotherapy tools against AD using antibodies targeting either the middle or C-terminal part of Aβ oligomers.

In summary, our study provides new data on the immunogenicity of small-sized Aβ1-42 oligomers and demonstrates their capability to induce antibodies directed predominantly to the N-terminal region of Aβ protein.

## Conclusions

Small Aβ1-42 oligomers of size 1–2 nm induce the strongest immune response in mice. The N-terminus of Aβ1-42 oligomers represents an immunodominant region which indicates its surface localization and accessibility to the B cells. The results of the current study may be important for further development of Aβ-based vaccination and immunotherapy strategies.

## Competing interests

The authors declare no competing interests.

## Authors’ contributions

ID was involved in the experimental design of the study, data collection, analysis and the manuscript drafting. RL generated the hybridoma. RB prepared and characterized the Aβ1-42 oligomers, GV set up the assay for the preparation Aβ1-42 oligomers, RM and VB contributed to the characterization of Aβ1-42 oligomers, AZ conceived of the study, drafted and edited the manuscript. All authors read, approved and contributed to the final version of the manuscript.
